# Lead Toxicity in Cereals: Mechanistic Insight Into Toxicity, Mode of Action, and Management

**DOI:** 10.3389/fpls.2020.587785

**Published:** 2021-02-04

**Authors:** Muhammad Aslam, Ayesha Aslam, Muhammad Sheraz, Basharat Ali, Zaid Ulhassan, Ullah Najeeb, Weijun Zhou, Rafaqat Ali Gill

**Affiliations:** ^1^Department of Plant Breeding and Genetics, University of Agriculture, Faisalabad, Pakistan; ^2^Department of Agronomy, University of Agriculture, Faisalabad, Pakistan; ^3^Zhejiang Key Laboratory of Crop Germplasm, Institute of Crop Science, Zhejiang University, Hangzhou, China; ^4^Queensland Alliance for Agriculture and Food Innovation, Centre for Crop Science, University of Queensland, Brisbane, QLD, Australia; ^5^Oil Crops Research Institute of the Chinese Academy of Agricultural Sciences/The Key Laboratory of Biology and GeneticImprovement of Oil Crops, The Ministry of Agriculture and Rural Affairs, Wuhan, China

**Keywords:** bioremediation, cereals, lead toxicity, mechanism, management, plant growth and development

## Abstract

Cereals are the major contributors to global food supply, accounting for more than half of the total human calorie requirements. Sustainable availability of quality cereal grains is an important step to address the high-priority issue of food security. High concentrations of heavy metals specifically lead (Pb) in the soil negatively affect biochemical and physiological processes regulating grain quality in cereals. The dietary intake of Pb more than desirable quantity *via* food chain is a major concern for humans, as it can predispose individuals to chronic health issues. In plant systems, high Pb concentrations can disrupt several key metabolic processes such as electron transport chain, cellular organelles integrity, membrane stability index, PSII connectivity, mineral metabolism, oxygen-evolving complex, and enzymatic activity. Plant growth-promoting rhizobacteria (PGPR) has been recommended as an inexpensive strategy for remediating Pb-contaminated soils. A diverse group of *Ascomycetes* fungi, i.e., dark septate endophytes is successfully used for this purpose. A symbiotic relationship between endophytes and host cereal induces Pb tolerance by immobilizing Pb ions. Molecular and cellular modifications in plants under Pb-stressed environments are explained by transcription factor families such as bZIP, ERF, and GARP as a regulator. The role of metal tolerance protein (MTP), natural resistance-associated macrophage protein (NRAMP), and heavy metal ATPase in decreasing Pb toxicity is well known. In the present review, we provided the contemporary synthesis of existing data regarding the effects of Pb toxicity on morpho-physiological and biochemical responses of major cereal crops. We also highlighted the mechanism/s of Pb uptake and translocation in plants, critically discussed the possible management strategies and way forward to overcome the menace of Pb toxicity in cereals.

## Introduction

Heavy metals (HMs) naturally exist in the Earth’s crust as soil constituents (Lasat, 2002). In the environment, HMs persistently accumulate due to stability and non-degradability. As a result of industrialization and anthropogenic activities (mining, electroplating and leather tanning, etc.), and disruptions in the natural marine and terrestrial habitats, this accumulation is growing rapidly (Meagher, 2000). Depending on their geological origin in the soil or human and industrial contamination, the concentration of HMs in soil can vary from 1 to 100,000 mg kg^–1^ (Blayock, 2000). For example, approximately 20 million hectares of agricultural land is irrigated with industrial effluents which contain traces of different elements as reviewed in Khalid et al. (2018).

These rapid changes in soil climate driven by anthropogenic activities override the natural adaptability of plants. Given the dependence of plants on soil media for acquiring essential elements for completing their life cycle, a high degree of versatility is needed to acclimatize the rapidly evolving conditions. Along with essential nutrients, plants uptake unknown toxic elements; some of them are HMs such as mercury, chromium, cadmium, arsenic, and lead (Pb). Heavy metals can enter into human body by consuming these contaminated foods and pose health risks (kidney diseases, renal tubular dysfunction, and bone damage) (Xie et al., 2018). Similarly, HMs disrupt physiological and biochemical processes in plants, such as photosynthesis, protein synthesis, redox balance, and energy transduction, as they accelerate generation of reactive oxygen species (Kopyra and Gwóźdź, 2003; Ali et al., 2015a, b). By disrupting the biological processes of plants, HMs may induce excessive micronutrient uptake and toxicity in plants (Peralta-Videa et al., 2009). There are several plant species that show tolerance to higher concentrations of HMs and toxic substances and are well adaptable to metalliferous soils, i.e., *Juncus effusus* (Kiran and Prasad, 2017; Najeeb et al., 2017).

Lead (Pb) is a very soft, dense, ductile metal with relatively low electrical conductivity than other metals. Lead is a nonessential element and when taken up by plants can negatively affect plant metabolic processes (Ali et al., 2014a, b). As it is an anthropogenic environmental pollutant, the crops cultivated on Pb-contaminated soils suffer poor germination, root growth, and biomass production. In nature, Pb forms minerals by interacting with other elements, and therefore is rarely present in native forms. The use of Pb for various purposes since ancient Rome is clarified by archeological findings. Whereas, its environmental concentration has risen more than 1,000 times over the last three centuries due to human interventions, with the largest rise from 1950 to 2000. Mineral deposits, along with copper and zinc, are typically the primary source of Pb. Globally, Pb contamination is expected to further increase due to its high application in the automotive and electric bicycle industries (Roberts, 2003). Urban agriculture, which relies on crop production in the vicinity of industries often cultivates food crops on polluted soils. Food demand is strong in urban areas, so the trend towards using polluted arable soils for cereal cultivation is rising (Peralta-Videa et al., 2009; Table 1).

**TABLE 1:** Lead production and reserves.

Country	Production (1,000 m^3^ ton)	Reserves	Country	Production (1,000 m^3^ ton)	Reserves
USA	400	7,000	Peru	280	6,000
Australia	620	27,000	Poland	35	1,500
Bolivia	90	1,600	Russia	90	9,200
Canada	65	650	South Africa	50	300
China	1,600	13,000	Sweden	65	1,100
India	95	2,600	Other	330	4,000
Ireland	45	600	**Total**	**4,100**	**80,000**
Mexico	185	5,600			

*Source: Geological Society of America (http://geology.com/usgs/lead/).*

In most parts of the globe, cereals are the main staple food and play a crucial role in global food security. The key goal to meet an ever-increasing demand for food for more than 9 billion people by 2050 is expected to increase pressure on grain industry (Akinyele and Shokunbi, 2015). Typically, cereals contain 75% starch, 15% protein, and 3% fat; they also contain vitamins (folate and tocotrienols), minerals, phytochemicals (lignans, alkylresorcinols, and sterols), and antioxidants in different concentrations (Ragaee et al., 2006). Micronutritional constituents, trace elements, and dietary fibers are rich in bran and cereal semen (65–85%, Akinyele and Shokunbi, 2015). Phytochemicals present in bran play a key role in reducing the risks of coronary diseases. Likewise, by modulating cellular oxidative status, the phenolic compounds of cereal grains protect the body from oxidative harm. Dietary fibers enhance food’s functional properties, such as viscosity, retaining capacity for water and oil, and swelling capacity. The adverse effects of high use of cereal-based dietary fibers are minimized by various processing technologies (microfluidization, grinding methods, thermal treatment, and bioprocessing) (Sen et al., 2020; Table 2). In most cereals, roots only penetrate into the soil from where the HMs are absorbed to a depth of 25 cm. The transfer of HMs to commercially valuable crop plants (particularly cereals) is of great concern, which can lead to biomagnification *via* entry into the food chain (Peralta-Videa et al., 2009).

**TABLE 2:** Contribution of cereals in energy consumed by humans.

Consumption or energy	World	Asia	Africa	South America	North America	Europe	Oceania
g capita^–1^ day^–1^	403	426	414	318	293	362	249
kcal capita^–1^ day^–1^	1,292	1,422	1,284	967	812	1,007	764

*Source: FAO. Food Consumption Database (FAO, 2013).*

## Lead Uptake Mechanism

In order to maintain nutrient homeostasis, plants must regulate nutrient uptake and respond to changes in the soil as well as within the plant. Plants utilize different strategies for mobilization and uptake of nutrients as well as chelation, transport between the various cells and organs of the plant and storage to achieve whole-plant nutrient homeostasis. Metal bioavailability is regulated by: (a) soil mineral status; (b) mineralization; and (c) membrane transporters. The following are the two major routes for plant metal uptake: (i) passive uptake, powered by membrane concentration gradient, and (ii) inducible substrate-specific and energy-dependent uptake (Williams et al., 2000).

A total of 17 different types of mineral elements needed for plants are transported to various organs according to their needs (Marschner, 2012). Many variables, such as soil physical and chemical properties, soil metal concentration, soil pH, redox status, and soil permeability play a direct or indirect role in the mobility of essential and nonessential elements in the soil (Dong et al., 2009). In the form of complexes, Pb is immobilized in the soil and thus becomes unavailable for plant uptake (Peralta-Videa et al., 2009). As it is a nonessential element for plants, there is no specific channel for Pb uptake. Plant species have different abilities and pathways for absorbing and transporting Pb ions. On root tips, this element is found within the soil as linked to carboxylic groups of mucilage uronic acids (Sharma and Dubey, 2005). Lead ions differently affect uptake of other essential nutrients i.e., it has a positive effect on Ca and K and negative effect on Mg absorption.

### The Rhizosphere, Root Exudates, and Element Uptake

As an active part of soil, the rhizosphere is directly affected by numerous organic compounds and root exudates (Jones et al., 2009). For example, 10–40% of the plants’ (organic and inorganic) photosynthetically fixed carbon is released in this part of the soil (McNear, 2013). Mucilage, sloughed off root cap, boundary cells, and exudates constitute the rhizodeposition. Mucilage assists in soil lubrication, root defense from desiccation, soil quality improvement, and nutrient uptake during root tip development (McNear, 2013). In the availability and absorption of metals by plants, the rhizosphere and soil microorganisms play a key role. By catalyzing the redox reaction, microbial activity alters the trend of HM uptake process (Dinh et al., 2020). The microenvironment formed by various discharged compounds contributes to the toxicity of HMs in nutrient absorption, including Pb as tested in Jitendra et al. (2017).

Root exudates consist of compounds of low and high molecular weight which are released actively and passively. Components of low molecular weight help to attract and use soil biota, which promote the rhizosphere through colonization or symbiosis on roots (Hawes et al., 2002). In inter- and intraplant interactions, root exudates play a role, helping to signal events (Kumar and Bais, 2012). The important micronutrients in metallic soils become accessible to plant roots due to the discharge of metal chelators. By improving their mobility and solubility, these chelators enhance the bioavailability of metals, and this is achieved by breaking the bonds between metals and soil particles (Rao et al., 2018).

Lead, widely available in the plant rhizosphere, has poor bioavailability for plants to be absorbed as it precipitates as sulfates and phosphates (Blaylock and Huang, 2000). Furthermore, the translocation of Pb is restricted by the roots, which accumulate maximum Pb contents. As recently reported by Li et al. (2020), the bioavailability of Pb depends on its concentration in the soil, the physical and chemical state of the soil, and the particular genotype of specific plant species. Lead mobility is strongly influenced by soil pH levels, i.e., increased mobility and Pb absorption has been recorded in low pH soils (3.9). Isotopes labeling techniques showed that more than 90% of Pb produced from ryegrass and wheat passed through the atmosphere (Alexander et al., 2006).

In the cell wall or in the form of extracellular precipitates on root tissues, much of the absorbed Pb occurs as bound to exchangeable ion sites (Sahi et al., 2002). For Pb movement under low concentration in roots, the endodermis acts as a partial barrier. Lead ions settle on the cell wall in the case of wheat roots and can be extracted by using citric acid (Xie et al., 2018). After getting into the root cortex, Pb ions transfer to the apoplastic space through the conductive transpiration system. Furthermore, it gives symplastic access to the region of lateral root initiation and young roots that grow. Its mobility is regulated by cross-membrane movement within the cytoplasm and membrane protein (Dong et al., 2009).

**FIGURE 1:**
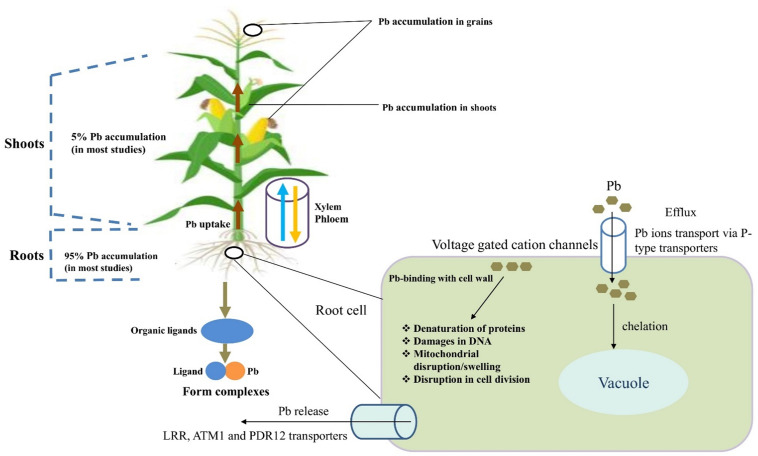
Potential uptake mechanisms and accumulation of lead (Pb) in plant parts. The sequence of Pb accumulation in different organs of cereals particularly in wheat is as; roots > shoots > shells > grains. Relatively higher Pb accumulation in root tissues could be an inactivate Pb in root cell walls. However, accumulation of Pb in grain is of main concern regarding its entry in food chain. Bioconcentration factor (BCF) and translocation factor (TF) are very important characteristics to be focused regarding accumulation and translocation of Pb. The heavy-metal mobility factor (MF) is equal to the ratio of the sum of labile fractions (exchangeable and bound-to-carbonate fractions) to the sum of all fractions, which is a good index to describe the relative mobility and bioavailable form of the metals in soils.

Transporters that are present in xylem parenchyma cells or phloem companion cells primarily facilitate the long-distance transport of Pb from roots to shoots (Dong et al., 2009). The transport of Pb occurs from the soil into root epidermal cells, followed by loading into the root xylem vessels for distribution into other plant organs. All plant transporter proteins are membrane proteins with Pb homeostasis as their responsibility (Romanowska et al., 2012). Vacuole sequestration potential (VSC) also plays a major role in the transport and sequestration of metals over long distances (Williams et al., 2000). *Via* mutual cooperation, tonoplast-localized transporters and ion chelators automatically change the available metal ions in the surrounding region (Peng and Gong, 2014; Figure 1).

Translocation/transport phenomena of Pb slightly differ from other HMs. Normally, 5% of Pb is transported into aerial components (Kiran and Prasad, 2017) and 95% remains accumulated in roots (Chandra et al., 2018). The possible mechanism is that in plant roots, ion-transferable cell wall sites mediate extracellular Pb binding. In this way, with the carboxyl group, Pb forms stable binding complexes and remains located in the roots. The components of the cell wall, such as glucuronic acid, limit apoplastic transport into the above plant components (Inoue et al., 2013). Lead efflux is regulated by P-type transporters or Pb cellular complexes that decrease the toxicity of Pb by endorsing sequestration into a specific organelle (Jiang et al., 2017). Previous studies have indicated that leucine-rich repeat (LRR), ATM1, and PDR12 the transporters may be associated with cell cellular Pb extrusion (Zhu et al., 2013).

## Toxicity Level and Mode of Action

Heavy metal is defined as “a metal with a density greater than 5 g cm^–3^.” Of a total of 53 HMs that occur naturally, 17 are biologically available and are important for the ecosystem (Alamri et al., 2018). For plants, Pb is a nonessential element, so it becomes toxic even at low concentrations. It is transferred from the atmosphere to plants quickly (Mishra et al., 2020). In cereal grains, the allowable Pb amount is >0.20 mg kg^–1^ (WHO/FAO, 2016). In response to varying Pb levels, genotypic variations are observed among species. For example, 30 ppm of Pb in the soil could reduce the production of maize genotypes by about 76 to 85% (Ghani et al., 2010). Maize plants translocate 2 to 5% of the soil absorbed Pb from roots to leaves. The root endodermis barrier may be the explanation for this small amount of translocation (Ghani et al., 2010). The low penetration capacity of Pb through the endodermis layer in maize was verified by historochemical observations (Sharma and Dubey, 2005). There is a weak bonding between Pb compounds and phytochelatins due to the large ion radius and high concentration number (Sharma and Dubey, 2005). The plants do not allow Pb to freely penetrate to the protoplast through cell wall tissues. Actively dividing cells are relatively susceptible to Pb concentration. Pb concentrations above 100 μM typically become toxic for callus induction (Krishania and Agarwal, 2012). In addition, high Pb concentrations in soil media minimize the supply of other plant uptake nutrients [Ca, Fe, Mg, Mn, P, and zinc (Zn)] by blocking entry or binding the ions to the ion carrier (Dong et al., 2009).

## Effect on Plants

In plants, three molecular mechanisms related to Pb toxicity are established on the basis of chemical and physical plant properties: (a) development of ROS, (b) blocking of essential functional groups in biomolecules, and (c) removal of essential metal ions from biomolecules. Incompletely filled Pb orbital present as cations in various physiological processes and can adversely affect seed germination rate, seedling growth, dry root shoot mass, efficiency of photosynthesis, mineral nutrition, and enzyme-related activity (Munzuroglu and Geckil, 2002). By accelerating the development of hydroxyl radical, Pb toxicity causes oxidative damage to plants (Pirzadah et al., 2020). Cereal crops respond differently to varying Pb toxicity levels and can be classified as metal excluders, metal indicators, and metal accumulators. Excluders avoid the accumulation and translocation of metal ions in their shoots; metal indicators accumulate metals in the aerial shooting system; metal accumulators absorb, translocate, and accumulate metal ions at levels greater than those found in the soil in various parts of their shoots (Ahmad et al., 2020; Zanganeh et al., 2020). Lead also affects plant growth by interfering with the composition and concentration of nutrients and protein conformation, like transporters and regulatory proteins (Pirzadah et al., 2020).

## Effect on Plant Growth and Development

**FIGURE 2:**
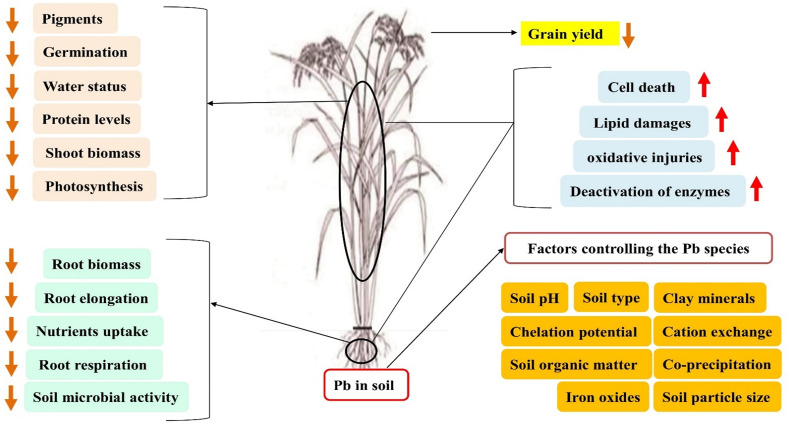
Thematic of Pb stress in plants. Excess quantity of Pb in the soil negatively impact on the soil microbial activity, root respiration, imbalance nutrient uptake, and overall morphology and biomass. Lead in upper plant parts such as stem and leaf deteriorates the photosynthetic machinery and protein level and disturb the water status and shoot and leaf biomass that resulted in the grain yield loss.

It is well known that Pb negatively affects germination, seedling vigor, growth of root and shoot biomass, photosynthetic activity, and enzymatic activity of plants (Munzuroglu and Geckil, 2002). For example, embryo energy generation is a critical germination process; reduction or blockage, along with the normal mitosis process, adversely affects protein, RNA, and DNA synthesis. The effects of Pb toxicity on seed germination are concentration dependent. These effects are more serious under the degree of increased and persistent toxicity (Figure 2). Crop germination that is specifically exposed to Pb toxicity in the soil is the first step of plant life. Toxic accumulation of Pb interferes with the formation of spindles and the growth of cell walls, decreasing cell expansion and dividing results in inhibition of root volume (Zulfiqar et al., 2019; Kanwal et al., 2020). During seedling growth and development, roots are more susceptible compared with shoots (Yang et al., 2010) because they are exposed to Pb toxicity. A well-known index of Pb toxicity tolerance is root growth potential. Under a high Pb toxicity level, plant growth is adversely affected due to poor nutrient uptake and transport (Ali et al., 2014b). However, lower concentrations of HMs, like Pb, often promote metabolic activities, and enzymes involved in these processes encourage growth and development.

## Effects on Plant Physiology

**FIGURE 3:**
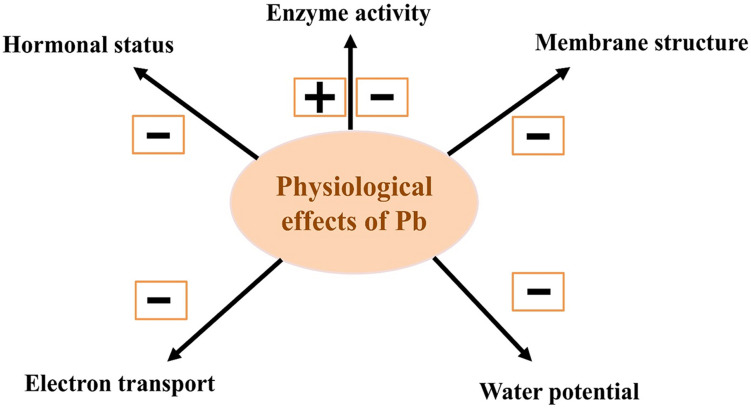
Lead toxicity results in overall disturbance in physiological mechanisms in plants. Here, “+” and “−” signs are positive and negative effects, respectively.

Plants exposed to various environmental contaminants have variable species and genotypic biological responses. Crops, such as cereals, are often exposed to HM toxicity and undergo a drastic reduction in grain yield. HMs can bind within cells with oxygen, nitrogen, and sulfur atoms; thus, HMs inactivate enzymes by binding with cysteine residues. HMs also induce lipid peroxidation, which leads to downregulation of peroxidases, and damages thylakoid membranes (Ali et al., 2014). Key genes of peroxidase family play a very important role in plant cell enzyme defense by searching for ROS during stress (Gülen et al., 2008). Lead toxicity accelerates development of ROS, including H_2_O_2_, which in turn causes secondary oxidative stress, leading to inhibition at the early stages of growth (Yang et al., 2010). When plants are facing the extreme levels of environmental stresses, the level of malonaldehyde (MDA) increased (depending upon the nature of stress). Malonaldehyde content represents the level of lipid peroxidation and its increased level acts as stress indicator. No improvement in MDA content under Pb toxicity was noted in germinating wheat seeds (Radić et al., 2010). In conjunction with other metals, Pb forms a complex combination to decrease the physiological efficiency of cereals. For example, high Pb and Cd concentrations substantially lower grain yield of rice. As complex in soil, HMs such as Pb, Cd, Cr, and Cu have a complex adverse effect on biochemical and physiological processes at various stages of rice growth and development (Xie et al., 2018; Figure 3 and Table 3).

**TABLE 3:** Lead induced phytotoxic effects on the morphological and physiological attributes of cereals.

Applied Pb dose	Plant specie	Culture	Exposure hours (h) or days	Alteration in plant parameters	References
250 and 500 mg kg^–1^ PbNO_3_	Mung bean	Hydroponic	21 days	↓ growth, photosynthetic pigments, protein synthesis, water use efficiency	Arif et al., 2019
100, 200, 300 μM PbNO_3_	Tartary buckwheat	Hydroponic	15 and 30 days	↓ shoot and root length and biomass, chlorophyll ↑ proline, soluble sugar, protein content	Pirzadah et al., 2020
418.64 mg kg^–1^ Pb	Rice	Hydroponic	23 days	↑ proline, soluble protein content	Rao et al., 2018
0, 50, and 250 μM	Wheat	Hydroponic		↑ radicle and coleoptile length	Kaur et al., 2015b
228 mg L^–1^	Rice	Hydroponic	8-16 days	↑ protein carbonylation, nonprotein thiols ↓ protein thiols	Srivastava et al., 2014
4,968 mg L^–1^	Wheat	Hydroponic	30 days	↓ growth traits, nutrients uptake	Lamhamdi et al., 2011
1,656 mg L^–1^	Rice	Hydroponic	30 days	↑ phosphorylation of PSII core proteins, protein degradation	Romanowska et al., 2012
1 mM	Rice	Hydroponic	4-7 days	↓ shoot or root length, biomass, nutrients uptake	Khan et al., 2018
0 and 100 μM	Rice	Hydroponic	7 days	↓ plant height, shoot, and root dry or fresh weights ↓ photosynthetic pigments, gas exchange attributes	Chen et al., 2017
0.5 and 1 mM	Wheat	Hydroponic	7 days	↓ plant growth, biomass, leaf relative water, chlorophylls ↑ proline	Hasanuzzaman et al., 2018
2 mM	Wheat	Hydroponic	63 days	↓ plant growth traits, biomass, leaf relative water, Rubisco activity, ATP sulfurylase, nutrients level, total chlorophyll	Alamri et al., 2018
1.5 mM	Wheat	Hydroponic	5 days	↓ root elongation and coleoptile growth	Turk et al., 2018
0, 1, 25, 50, 100, 200, and 500 mM	Maize	Hydroponic	14 days	↓ early growth, biomass, seed germination, total protein contents, Pb uptake	Hussain et al., 2013
500 mg kg^–1^	Wheat	Soil	120 days	↓ growth, biomass, Pb uptake, grain yield, chlorophyll, gas exchange parameters	Rehman et al., 2017
0.1 mM	Maize	Hydroponic	4 days	↓ growth rate of coleoptile segments ↑ membrane hyperpolarization	Kurtyka et al., 2018
0, 200, 400, 800, 1,600, and 3,200 mg kg^–1^	Sorghum	Soil	21 days	↓ growth, dry matter, photosynthetic rate, transpiration rate, starch, proteins, total soluble sugars	Cândido et al., 2020

### Plant Growth Regulators

Plant growth regulators (PGRs) are naturally occurring organic compounds that under normal and stressful regimes, play a vital role in plant growth and development (Bajguz and Piotrowska, 2009; Davies, 2010; Piotrowska and Bajguz, 2011; Ahanger et al., 2018; Šimura et al., 2018). These PGRs are auxins, cytokinins, brassinosteroids (BRs), gibberellins, ethylene, abscisic acid, and jasmonic acid (JA). Out of these, BRs belong to a group of phytohormones which controls various biological processes, including HMs and broad range of environmental stresses. They activate the transcription factor coding gene [BRASSINAZOLE RESISTANCE 1 (BZR1)/BRI1-EMS SUPPRESSOR 1 (BES1)], which triggers thousands of BR-responsive genes. In addition, BRs modulate many physiological activities such as photosynthetic functioning (i.e., chlorophyll content and photosynthetic capacity), antioxidant enzyme activities, and metabolism of carbohydrates and mitigate the negative effects of HMs on plants (Anwar et al., 2018). Also, at a very low concentration, BRs function efficiently and alleviate the toxic effects of Pb stress. For example, through exposure to HMs, plant biosynthetic pathways are severely disrupted. One of the main detoxification mechanisms is the upregulation of cytochrome P450s under heavy metal conditions. Many biosynthetic pathways, such as jasmonic acid (JA), isoflavonoids, flavonoids, anthocyanins, coumarins, salicylic acid (SA), phytoalexins, and many others, play an important role in the metabolism of cytochrome P450s.

Other PGRs such as JA and SA (o-hydroxybenzoic acid) also relieve a range of environmental stresses, like HMs, including BRs (Wasternack, 2014; Liu et al., 2016). Jasmonic acid is typically present in higher plants and takes part in essential plant growth and development functions. Its production in plants begins (along with its methyl ester called methyl jasmonate) after exposure to biotic/abiotic stresses (Mohamed and Latif, 2017; Ashry et al., 2018). It also regulates HM toxicity and increases plant growth by modulating gene expression, osmolytes, antioxidant machinery, and carotenoids production (Creelman and Mullet, 1995; Poonam et al., 2013). For example, it improves growth, photosynthetic efficiency, nutrient uptake (i.e., N, P, and K), electrolyte leakage, and antioxidant machinery in Pb-stressed maize plants (Sofy et al., 2020). In comparison, SA is a phenolic acid usually found in plant organisms (Liu et al., 2016). Exposure to Pb is considered to have severely affected maize plants, reducing growth, yield, photosynthetic pigments, and mineral nutrients (nitrogen, phosphorus, and potassium) and increased electrolyte leakage (EL), accumulation of malondialdehyde (MDA), osmolytes, and nonenzymatic and enzymatic antioxidants (Rahmani et al., 2015; Liu et al., 2016; Hafez and Seleiman, 2017). As a hormone-like substance, it modulates physiological processes such as flowering, photosynthetic machinery, seed germination, plant growth, membrane permeability, and plant defense response against biotic and abiotic stresses. A group of researchers recently investigated that SA together in combination of JA effectively reduced the negative effects of Pb stress in maize (Sofy et al., 2020).

### Antioxidant System

The lower reactive oxygen species (ROS) level is needed for the functioning of cells and induces oxidative stress in excessive amounts. The excess amount of ROS was deemed harmful to the growth and production of plants. A physiological equilibrium between ROS formation and the defensive antioxidant function of the cell is ensured under normal conditions. Reactive oxygen species attack macromolecules (polyunsaturated chloroplast membrane fatty acids) which leads to lipid peroxidation acceleration (Ledford and Niyogi, 2005). In foods, lipid peroxidation results from rancidity and unpleasant odors/tastes. Superoxide and hydrogen peroxide (H_2_O_2_) are the main active oxygen species. Hydrogen peroxide has a long life and the ability to diffuse across membranes, so it is considered the second messenger of signals produced by ROS. In the sense of oxidative stress, there are many environmental stresses involved. One of them is Pb toxicity, which causes many enzymes to be inactivated by binding with their SH group, resulting in overproduction of ROS (Vinocur and Altman, 2005). These mutually interactive processes have a detrimental effect on cell structure and metabolism, resulting in a decreased performance of oxidation-reduction enzymes and electron transport systems. The changes in morphological, physiological, biochemical, and molecular processes are due to secondary (osmotic or oxidative) stresses that disturb membrane protein stability and interfere with cellular homeostasis (Ashraf, 2009).

Several enzymatic antioxidants, including super oxide dismutase (SOD), peroxidase (POD), catalase (CAT), ascorbate peroxidase (APX), and glutathione reductase (GR), as well as nonenzymatic antioxidants such as reduced glutathione (GSH), oxidized glutathione (GSSG), and ascorbic acid (AsA), play a significant function on their own and in combination in different plant growth processes to alleviate the adverse effects of ROS. For example, increased SOD and CAT activity was reported in maize at the germination and seedling stage when subjected to Pb stress (Zhang et al., 2018). In another study, AsA improved photosynthetic machinery and osmolyte activity; it also improved antioxidant protection systems in maize under HM stress (Zhang et al., 2019). Under extreme toxic conditions, plant protection mechanisms cannot be sustained at an acceptable level by endogenous regulators, including antioxidants. So, exogenous application of growth enhancers to mitigate the negative impacts of stresses are becoming widespread in many plants (Zulfiqar et al., 2019). For example, Pb stress improves the ROS in wheat, including the rate of H_2_O_2_, O_2_^–^, and lipid peroxidation. Exogenous supplementation of GSH greatly alleviates the redox reservoir of antioxidants and raises antioxidant activity but reduces the amount of ROS. In addition, exogenous GSH improves the quality of proline, balances the water status, prevents chlorophyll degradation, thus increasing plant growth and production and overall biomass (Hasanuzzaman et al., 2018).

### Photosynthesis

Higher Pb concentration interferes with physiological and biochemical processes involved in plant growth and development based on photosynthesis as photosynthesis is one of the processes most affected (Gomes, 2011). The toxicity of Pb induces changes in glycolipids, particularly in monogalactosyldiacylglycerol, which is associated with chloroplast membrane permeability (Rizwan et al., 2018). Lead toxicity causes an increase in stomatal resistance and a reduction in the rate of transpiration and photosynthesis. Stomatal conductance, chlorophyll content, and photosynthetic rate are decreased by increased Pb uptake by plants (Rasool et al., 2020). With the rise in the amount of Pb toxicity, a sharp decrease in chlorophyll content was observed (Ali et al., 2014b). The detrimental effect of Pb on the photosynthetic apparatus, enzymatic activities, and mineral nutrition is due to the strain on the physiological processes under Pb toxicity (Tian et al., 2014). Higher Pb concentration in plant tissues substitutes Mg with Pb which inhibits enzymatic activity and electron transport in Calvin cycle and restrict chlorophyll synthesis. The toxicity of Pb reduces the number of cristae in mitochondria, which have a negative effect on photosynthesis and respiration by reducing oxidative phosphorylation potential (Mroczek-Zdyrska and Wójcik, 2012).

### Energy Metabolism

Lead toxicity exerts adverse impacts on respiratory activities and ATP concentrations. There is an evidence that Pb toxicity impairs leaf respiratory activities. Lead toxicity enhances mitochondrial respiration with no effect on photorespiration, resultantly, there is an increase in respiratory process. Increase in respiration rate under Pb stress has association with ATP production in mitochondria; this energy is used for survival under Pb toxicity. Both C_3_ and C_4_ plants, when exposed to Pb toxicity experience 50% increase in respiration rate and fully oxidized substrates in mitochondria. Electron transport chain reactions are disturbed by Pb stress, as Pb bind with mitochondrial membrane causing decoupling of phosphorylation process (Romanowska et al., 2002; Ashraf and Tang, 2017). High Pb accumulation in plant starts NAD^+^-malate dehydrogenase activity, interrupts hill reaction, and interferes with cyclic and noncyclic photophosphorylation (Romanowska et al., 2002). Damage to secondary structures of PSII, blockage of energy transfer pathways among amino acids, and reduction in absorption of visible light are the result of high Pb intake. Under Pb stress environment, swelling of mitochondria, vacuolization of endoplasmic reticulum and dictyosomes, and damage to cristae occur. Lead toxicity significantly inhibits guard cells, cell wall elasticity, chlorophyll development, and leaf development which results in plant growth reduction and abnormal plant functioning (Kurtyka et al., 2018).

## Effects on Biochemical Processes

Plant growth and development is adversely affected by Pb toxicity. To overcome oxidative damage caused by Pb toxicity, several reactions have been produced by the plant defense mechanism, and various techniques are used to cope and adapt. This adaptability depends entirely on the difference in the relative protein abundance of stress-responsive protein; this fully changes the levels of proteome, transcriptome, and metabolome (Cho et al., 2008). In addition to the post-translational regulatory mechanism including protein degradation and RNA stability, the strength and length of the imposition of Pb toxicity affect the expression pattern and transcription level of these proteins, which have a further role in making the response of the plant more complex. The recent advances in interactome analysis, transcriptome and metabolome clarify in a better way the plant response to stress (Sharma et al., 2013). Such studies further promote understanding of the physiological and molecular pathways involved in Pb stress responses. The typical response to all stresses is mainly oxidative stress induction and gene expression modulation (Shri et al., 2009). Metals such as Pb play a very important role in plant enzymatic behavior. With the displacement of one metal with another, these activities are badly affected, e.g., divalent cations (CO^++^, Ni^++^, Zn^++^) displace Mg^++^ in ribulose-1,5-bisphosphate-carboxylase/oxygenase causing reduced enzymatic activity (Table 4). Lipid peroxidation is a biochemical marker for an injury mediated by free radicals. Rising the toxicity of Pb increases the peroxidation of lipids that causes oxidative stress. A similar response is observed in the case of glutathione reductase activity, with a higher level of Pb concentration decreasing catalase activity in roots and shoots (Ali et al., 2014b).

**TABLE 4:** Lead-induced phytotoxic effects on the oxidative markers and biochemical and metabolic traits of cereals.

Applied Pb dose	Plant specie	Culture	Exposure hours (h) or days	Alteration in plant parameters	References
0,100, 200, and 300 μM	Tartary buckwheat	Hydroponic	15 and 30 days	↑ H_2_O_2_, membrane stability index, GSH contents ↑ SOD, POD, CAT, GR, GST activities	Pirzadah et al., 2020
418.64 mg kg^–1^ Pb-contaminated soil	Rice	Hydroponic	23 days	↑ SOD, CAT, APX. Activities ↓ MDA, endogenous Pb contents	Rao et al., 2018
0,10, and 50 μM	Rice	Hydroponic	2 and 4 days	↑ SOD, APX, GR activities, MDA, α-tocopherol content ↓ CAT activity	Thakur et al., 2017
0, 16, 40, and 80 mg L^–1^	Maize	Hydroponic	8 days	↑ MDA, H_2_O_2_, O^–^._2_, SOD, APX, GPX, GR activities ↓ CAT activity,	Kaur et al., 2015a
0, 50, and 250 μM	Wheat	Hydroponic	1 days	↑ MDA, H_2_O_2_, O^–^._2_, conjugated diene, membrane damage ↑ SOD, CAT, APX, GPX, GR activities	Kaur et al., 2015b
0, 16, 40, and 80 mg L^–1^	Maize	Hydroponic	3, 12, and 24 h	↑ MDA, H_2_O_2_, thiols, APX, DHAR, MDHAR activities ↓ AsA, GSH contents	Kaur et al., 2015c
0, 50, 100, 250, and 500 μM	Wheat	Hydroponic	4 days	↑ SOD, CAT, MDA ↓APX, GPX, GR activities	Kaur et al., 2013
0, 50, 100, and 200 μM	Rice	Hydroponic	16 days	↑ SOD, MDA ↓CAT, POD activities	Li et al., 2020
0, 1, 2, and 4 mM	Wheat	Hydroponic	3 days	↑ SOD, CAT, POD, APX activities, MDA	Yang et al., 2011
0, 0.15, 0.3, 1.5, and 3 mM	Wheat	Hydroponic	6 days	↑ SOD, POD, APX activities, MDA ↓ CAT activities	Lamhamdi et al., 2011
0, 500, 1,000, and 2,500 μM	Wheat	Hydroponic	7 days	↑ SOD, GPX activities, MDA	Kaur et al., 2012
228 mg L^–1^	Rice	Hydroponic	days	↑ SOD, GPX activities, MDA	Srivastava et al., 2014
0-200 μM	*Sedum alfredii*	Hydroponic	14 days	↑ SOD activities ↓ APX activities	Gupta et al., 2010
1 mM	Rice	Hydroponic	18 days	↑ SOD, POD activities, MDA, H_2_O_2_, OH^–^, O^–^_2_ levels	Khan et al., 2018
0 and 100 μM	Rice	Hydroponic	7 days	↑ SOD, GR, APX activities, AsA, G.S.H., H_2_O_2_, O^–^_2_ levels	Chen et al., 2017
0.5 and 1 mM	Wheat	Hydroponic	7 days	↑ MDA, H_2_O_2_, O^–^._2_ methylglyoxallevels, SOD, GST activities ↓AsA-GSH content, CAT, GPX, GR, glyoxalase system enzymes	Hasanuzzaman et al., 2018
2 mM	Wheat	Hydroponic	63 days	↑ MDA, H_2_O_2_, cysteine levels, SOD, CAT, GR activities	Alamri et al., 2018
1.5 mM	Wheat	Hydroponic	5 days	↓ AsA-GSH content, CAT activity ↑ MDA, H_2_O_2_ content, SOD, GPX, GR, APX. Activities	Turk et al., 2018
0, 200, 400, 800, 1,600, and 3,200 mg kg^–1^	Sorghum	Soil	21 days	↑ SOD, CAT, APX activities, MDA, H_2_O_2_ contents	Cândido et al., 2020

## Lead in Food Chain

Lead acts as a physiological and neuronal toxin and is naturally carcinogenic. In humans, the absorption of Pb is by the ingestion of paint chips, vehicle pollution inhalation, Pb-welded canes, and water intake. Due to the capability of rapid bioaccumulation, lower degradability rate and long biological half-life, Pb is of great concern in the food safety-security (FSS) debate (Ayesha et al., 2019). When Pb is consumed, the half-life of long excretion is considered to be continuous (in order of years). Contamination of HMs in marine areas results in the accumulation of HMs in aquatic body tissues. Subsequently, this bioaccumulation is biomagnified (grazer → primary consumer → secondary consumer → top predator) to various levels. Ultimately, by taking polluted sea food, humans are affected. Pregnant women are more vulnerable to HM toxicity because of the trans-placental metal change in maternal blood. Individuals with chronic exposure to Pb toxicity suffer from memory loss, poor nervous system functioning, decreased understandability, decreased blood pressure, kidney and brain damage, adverse effects on intellectual ability, and sluggish reaction by taking more time to respond. Men’s exposure to Pb toxicity cause damage to the responsible organs of sperm production, raises the amount of Pb in the blood, and the risk of death.

**FIGURE 4:**
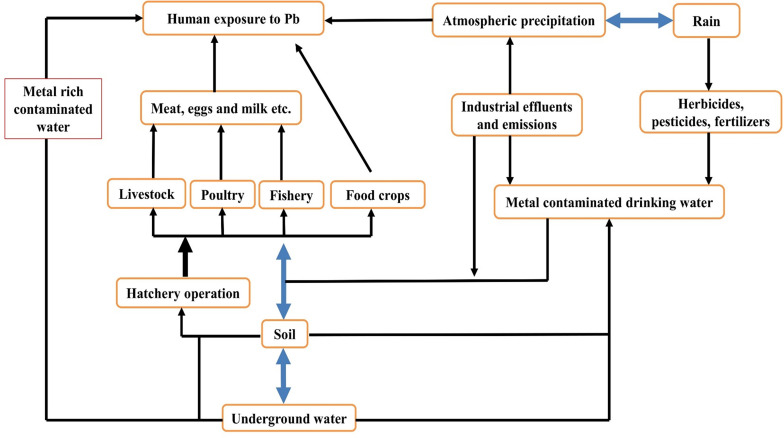
Pathway of terrestrial ecosystem through which humans are exposed to Pb toxicity.

It is well known that high soil accumulation of Pb is a possible hazard to all soil-related lives (Zhuang et al., 2009). The soil state is frequently undocumented and thus, exposed to high levels of harmful compounds unintentionally. Individuals who eat food cultivated on polluted soils can be at risk of toxic health effects. Lead toxicity in pregnant women causes newly born babies to have low birth weight and intellectual disability. Central nervous system destruction, neurological disease and cancer of various body organs are most of the recorded effects of Pb toxicity (Uwah et al., 2011). Lead accumulation should not be overlooked in particular organs of living organisms (Zhuang et al., 2009). Two known binding polypeptides responsible for Pb binding in the kidneys are thymosin beta 4 and acyl-CoA. Pb binds with low molecular weight compounds like sulfhydryl groups in the blood serum. Exposure to Pb toxicity suppresses Zn-superoxide dismutase activity in humans under zinc deficiency, resulting in oxidative stress in the kidneys, blood, and liver; this disorder may be treated by normal Zn intake (Chemek et al., 2016). The absorption, translocation, metabolism, and retention of Pb in the body are limited by zinc supplementation. Good practices relating to agriculture should be enforced, taking into account the health issues of humans related to farming on polluted soils. Farmers should be informed about the health risks associated with the imbalance of the use of pesticides, fertilizers, and wastewater as irrigation through various official programs and media broadcasts (Uwah et al., 2011; Figure 4 and Table 5).

**TABLE 5:** Concentration of lead in cereal grains.

Crop	Country	Study/Contamination	Pb contents (mg kg^–1^)	Crop	Country	Study/Contamination	Pb contents (mg kg^–1^)
Barley	Ethiopia	Market survey (Tegegne, 2015)	0.03	Rice	France	Market survey (Leblanc et al., 2005)	0.01
Barley	Ethiopia	Farmer field (Eticha and Hymete, 2015)	0.82–5.64	Rice	Nigeria	Market survey (Akinyele and Shokunbi, 2015)	<0.08
Maize	Nigeria	Contaminated soil (Orisakwe et al., 2012)	1.01	Rice	India	Organic farming (Chandorkar and Vaze, 2013)	0.10
Maize	Nigeria	Market survey (Akinyele and Shokunbi, 2015)	<0.08	Rice	Australia	Market survey (Rahman et al., 2014)	0.02–1.30
Maize	Bangladesh	Field survey (Islam et al., 2014)	0.04–1.30	Rice	India	Peri-urban areas (Tripathi et al., 1997)	0.02
Millet	Finland	Market survey (Ekholm et al., 2007)	0.02	Rice	Bangladesh	Field survey (Islam et al., 2014)	0.07–1.30
Millet	Nigeria	Contaminated field (Orisakwe et al., 2012)	3.54	Sorghum	Ethiopia	Market survey (Tegegne, 2015)	0.08
Oat	Finland	Market survey (Ekholm et al., 2007)	0.05	Sorghum	Bulgaria	Contaminated area (Angelova et al., 2011)	10.30
Rice	Nigeria	Farmer field (Orisakwe et al., 2012)	61	Wheat	India	Peri-urban area (Tripathi et al., 1997)	0.02
Rice	Saudi Arabia	Market survey (Othman, 2010)	0.02–0.03	Wheat	Ethiopia	Market survey (Tegegne, 2015)	0.05
Rice	Saudi Arabia	Market survey (Ali and Al-Qahtani, 2012)	6.16	Wheat	Saudi Arabia	Market survey (Ali and Al-Qahtani, 2012)	2.80
Wheat	Belgium	Organic and conventional farming (Harcz et al., 2007)	0.04–0.10	Wheat	Bangladesh	Field survey (Islam et al., 2014)	0.03–1.30
Wheat	Brazil	Market survey (Santos et al., 2004)	<0.01–0.02	Wheat	Nigeria	Market survey (Akinyele and Shokunbi, 2015)	<0.08
Wheat	Spain	Samples from flour industry (Tejera et al., 2013)	0.04	Wheat	India	Organic farming (Chandorkar and Vaze, 2013)	0.12
Maximum permissible limit			0.20				0.20

## Phytoremediation

Since they are not chemically degradable, most HMs, like Pb, have the ability to remain in the soil for thousands of years. Lead toxicity poses many health problems directly or through plant transport to higher species. The alternative is to physically extract it or convert it into nontoxic compounds (Tangahu et al., 2011). With regard to phytoremediation, the processes involved in nutrient absorption are of great significance, as the same applies to the uptake of toxic elements. In metal accumulating pathways in plants, phytoremediation technology for complex site decontamination has gained recent attention. With low cost and environmental protection, this approach is very evolving (Antonkiewicz and Para, 2016).

Plant absorption mechanisms associated with phyto-remediation technology can be grouped into organic and inorganic pollutants such as phytovolatilization, rhizo-filtration, phyto-stabilization, phyto-degradation, and rhizo-degradation (Tangahu et al., 2011). The method of rhizo-filtration is effectively carried out by young seedlings in ventilated water. A known remedy that works by reducing mobility and phyto-availability in the soil is HM immobilization by using modifications. In the case of Pb in polluted soils, the immobilization technique works well because of its adsorption capacity and biologically less accessibility compared with other toxic metals such as Cd and Cr (Dong et al., 2009). As a Pb remedy strategy, phytoextraction is known to be a good alternative to traditional techniques based on engineering; this solution is low cost, environmentally sustainable, and easily applicable. Various forms of phyto-remediation are defined as follows:

### Microbial Remediation

There are important economic and ecological benefits from the use of microbes to adjust the concentration of HMs in the soil and to boost the ability of plants to cope with high concentrations of metals (Alamri et al., 2018; Jin et al., 2018). The response of cereals to Pb toxicity can be improved by applying plant-associated microorganisms that promote growth and developmental physiology. The goal of this approach should be to create a plant-mycorrhizal association that is specifically tolerant of HM stress Pb toxicity (Glick, 2010).

#### Bacteria

Under unfavorable conditions, species that have a strong relationship with plants may be used to enhance plant growth. Inoculation of bacteria increases plant responses to stresses caused by HMs. These microorganisms are capable of fixing atmospheric nitrogen, solubilizing phosphate, and developing growth-promoting compounds, e.g., aminocyclopropane-1-carboxylate deaminase (ACCD), phyto-hormones, or siderophores. ACCD modulates ethylene production at plants (Belimov et al., 2005). Among the plant growth-promoting rhizobacteria (PGPR), some bacterial strains can synthesize auxins to enhance root growth resulted in the improvement in the plant growth and development (Hussain et al., 2013; Ahmed and Hasnain, 2020). Auxin producer bacteria physiologically enhance responses to toxicity of HMs in maize (Cassan et al., 2009). Chelating compounds produced by bacteria acidify the soil, making it possible to reach metals in the soil (Lasat, 2002). The bacterial ACC deaminase degrades ACC (ethylene precursor) and the development of rescue plants under HM stress (Mayak et al., 2004). In the defense of plant cell damage, bacterial catalases and oxidases play a very important role; bacterial catalases are very important for plant pathogenesis (Guo et al., 2012).

Plant growth-promoting rhizobacteria increases HM phytoextraction by promoting plant growth under Pb stress conditions. The trace elements can be immobilized in soils during phyto-extraction, resulting in reduced uptake (Glick, 2010). The main characteristics of phyto-stabilization are high soil-retaining ability and high tolerance to Pb toxicity. Bacterial isolates with good PGP characteristics in cereals help to increase cereal growth not only under normal soil conditions but also under stress conditions of HM. The goal should be to classify and characterize bacterial strains that have a role in improving HM tolerance, including Pb toxicity in cereals.

#### Fungi

Besides other microorganism’s, several strains of fungi are known to date that potentially involve in the phytoremediation of HMs in various plant species. For instance, recently, Manzoor et al. (2019) reported five nonpathogenic fungal strains, i.e., *Trichoderma harzianum*, *Penicillium simplicissimum*, *Aspergillus flavus*, *Aspergillus niger*, and *Mucor* spp. and recorded their ability to modify soil properties including pH and organic matter. They found that these above five strains significantly enhanced the bioavailability of Pb under different concentrations. Biogeochemical cycling of metals is correlated with microorganisms; their actions finalize the mobilization or immobilization of metals depending on the microenvironment and process involved (Ehrlich et al., 2015). Symbiosis between indigenous and plant microbes (indigenous and plant microbes) reacts to HM toxicity tolerance. The supply of heavy metals and their toxicity to plants depends on rhizospheric reactions and microbial activity. Various plant roots have strong symbiosis with mycorrhiza; this relationship significantly changes plant responses to various types of stresses of HMs (Ehrlich et al., 2015). The access of heavy metals to roots is limited by a fungal sheath around the root surface. In contrast to nonmycorrhizal roots, greater concentrations of soluble phenolics are present in mycorrhizal roots. Mycorrhizal fungi improve protection through the interaction of GSH mycorrhizal roots that act as a strongly armed relationship with physiological defense against Pb toxicity (Tangahu et al., 2011). This is a successful strategy for establishing associations of stress tolerant plant mycorrhizal for phytoremediation and reclamation and enrichment of soils. *Exophialapisciphila* (dark septate endophyte) increases the growth of maize and biomass under polluted metal soils and by regulating root to shoot translocation, it further enhances resistance to HM toxicity (Tangahu et al., 2011).

### Plant Engineering

The application of phytoremediation on a large scale, especially in the context of the soil-crop system, is often limited. Other issues exist, such as phytovolatilization, the issue of biomass disposal and the use of various chemicals for remediation. Using gene modification to enhance the resistance of HMs in food crops may overcome these limitations (Ashraf, 2009). This may be useful in enhancing the capacity for plant tolerance and contact between plants and microbes. Plant genetic engineering study to resolve the issue of susceptibility to HMs is a guarantee to increase the phytoremediation ability of polluted HM soils (Vinocur and Altman, 2005).

### Advantages and Limitations

Phytoremediation technology looks effective for the purpose, compared with various traditional techniques used to remediate HMs, including Pb-polluted soils. There are various forms of advantages and disadvantages (Table 6) associated with the phytoremediation process applied to Pb toxicity (Tangahu et al., 2011).

**TABLE 6:** Advantages and limitations of phytoremediation mechanism in Pb toxicity.

Advantages	Limitations
Applicable to different contaminants	Requires more root surface area and depth for efficient working
Low cost bearing as compared with traditional processes	Long-term commitment because of less biomass production due to slow root growth in Pb-contaminated soils (time consuming)
Efficiently reduces contaminant	Efficiency effected with the increasing age of plants
Less disruptive as compared with physical removal and chemical treatments	Survival of plants under variable Pb toxicity
Environment friendly	Variable climatic conditions adversely affect the working efficiency of plants
Esthetically pleasing	Variable soil chemistry
Easy monitoring	Pb bioaccumulation in plants and its transportation to plant tissue.
Possibility of recovery of different metals	Availability of contaminant for primary consumer through food chain
Reuse of metals (phyto-mining)	No assurance of complete removal of contaminant from soil

## Phyto-Management

By following the process of avoidance, tolerance, or both, protection from HM stress can be achieved. In detoxification, tolerance functions better than selective absorption. The most desirable characters in plants involve high Pb accumulation in roots than shoots and strong defense mechanism. Plants with the ability to prevent metals from entering the cytoplasm are known as avoiders, while plants with the ability to detoxify metal ions with the ability to cross plasma/organellar membranes are labeled as tolerant (Ledford and Niyogi, 2005). Mobile and immobilized fractions of HMs need to be separated as they bind to organic and inorganic soil components and to humus. Solubility, availability, and mobility depend entirely on the characteristics of various soil types, such as adsorption, desorption, and complexation (Kaur et al., 2015c). With a priority on food security for a rapidly increasing population, the production of nonfood crops in metal-polluted soils is not feasible. These soils can be used for food crops by adding various modifications to reduce the absorption of metals by plants. Some of the changes are clarified below.

### Inorganic Amendments

For enhancing Pb tolerance in cereals, safe and proper nutrition for plants could play an effective role. As a micronutrient, sulfur plays a role in plant protein synthesis and is part of several coenzymes and prosthetic groups as a structural unit. Sulfur (S) improves the accessibility of metals to plants by reducing soil pH (Cui et al., 2004). Zn fertilization increases the yield of grain in cereals by decreasing the negative effect of Pb toxicity due to its antagonistic effect on soil uptake (Dalir et al., 2017). This antagonistic interaction works positively and causes Pb uptake to be decreased, since Pb uses Zn membrane transport proteins for membrane mobility for uptake (Mousavi et al., 2012). Thus, the use of Zn fertilizer acts to compensate for Zn deficiency and mitigate the impact of Pb toxicity. In iron assimilation and eventually chlorosis, excessive Zn concentration induces blockage.

Silicon (Si) application increases plant growth and biomass under HMs. Under HM stress conditions, it improves photosynthetic machinery (chlorophyl concentrations, gas exchange capacity, and fluorescence in cereals) (Rizwan et al., 2019). Silicon deposition increases maize resistance to HM toxicity in root endodermis and pericycle. The application of chelants, EDDS, EDTA, and IDSA could increase the concentration of Pb, Cu, and Cd in hydroponics maize shoots (Zhao et al., 2010). The positive effect of diammonium phosphate (DAP) on root weight and grain yield in cereals is important. This improvement is documented as a result of Pb immobilization and its role in improving Pb toxic effects on plant growth (Park et al., 2012). In case of wheat, diammonium phosphate (DAP) effectively decreased Pb concentrations in roots, straw, and grains; this may be attributed to increased soil pH and decreased Pb mobility (Rehman et al., 2015). Diammonium phosphate decreases soil extractable Pb concentration due to the formation of secondary Pb phosphate precipitates (Miretzky and Fernandez-Cirelli, 2008). Diammonium phosphate increases P concentration in alkaline soils, resulting in the formation of phosphate-induced fluoropyromorphite and other compounds with low soil surface adsorption, solubility, and complexity (Chen et al., 2009). It also improves the photosynthetic rate effectively. Amendments that contain phosphorus and calcium allow stable metal compounds and reduce their mobility and phyto-availability (Rehman et al., 2015). Inorganic nutrients are considered the key ingredient of MS media and the effect on plants is the best variable to study. Magnesium (Mg) and zinc (Zn) improve the efficacy of callus growth and regeneration. For instance, Zn nanoparticles alleviate the negative impacts of abiotic stress in plants and enhance callus regeneration rate, improving the mineral elements (N, P, and K) and enzymatic antioxidants such as SOD and GPX (Alharby et al., 2016). In another study, researchers studied the role of inorganic nutrients such as Mg (MgSO_4_) and Zn (ZnSO_4_) to alleviate the toxic levels of HMs (Pb and Ni) for callus induction grown on regeneration medium (MS). They reported the varied response of inorganic nutrients to both HMs and noted that callus induction rate was significantly deteriorated when grown in MS medium containing more than 100 μM concentrations of Pb and Ni. They also concluded that ZnSO_4_ was best to minimize the Ni stress and MgSO_4_ was best against Pb toxicity (Krishania and Agarwal, 2012). These above-mentioned studies suggested that regarding phytoremediation, the long-term use of single fertilizer should be discouraged. In combination, different inorganic additions, fertilizers, and agronomic practices could be more beneficial.

### Organic Amendments

A selection of farm yard manure can achieve good phytoextraction efficiency in cereals (FYM). Organic matter plays an important role in decreasing metal phyto-availability, providing additional nutrients and changing the physical, chemical, and biological properties of the soil with decomposition (Rizwan et al., 2016). Gypsum-released calcium ions in soil compete with Pb for plant uptake, in addition to PbSO_4_ since Pb precipitation may be another mechanism of action (Arnich et al., 2003). Application of organic amendments decreases toxicity of HMs and enhances plant growth (Mohamed et al., 2015). Under field conditions, its implementation increases root branching and surface area and fine roots. In maize, the biochar application changes the root architecture and influences the absorption of HMs. The response of cereals varies with the change in organic modification type, growth conditions, and the degree of toxicity of HMs in soil (Al-Wabel et al., 2015). The treatment of metal contaminated soils with dihydrazone facilitates the absorption by maize plants of Zn, Pb, Cu, and Cd (Antonkiewicz and Para, 2016). Organic acids with low molecular weight (LMWOA) play a role in reducing shoot biomass and HM concentration in maize shoots (Sabir et al., 2014).

By minimizing Pb absorption, the application of different modifications improve physiological activity and natural chlorophyll synthesis, all of which help to normalize the transport of electrons and enzymatic activity in the Calvin cycle (Mroczek-Zdyrska and Wójcik, 2012). These amendments release soil nutrients that encourage root growth and development and increase the site’s microbial activity. Organic modifications are solar-based, cost-effective, and eco-friendly methods that preserve natural soil properties (using plants and microbes). The use of plants such as *Erigeron canadensis*, *Digitari aciliaris*, and *Solanum nigrum* can mitigate the carcinogenic potential of Pb as bioremediation (Al-Wabel et al., 2015).

### Source Reduction

The reduction of HM sources is an important technique for minimizing its dangerous impact. The accumulation of HMs in soil and eventually in food crops could be minimized by an attempt to control air quality. Compared with humans fed food raised under regular irrigation, the content of HMs in humans fed with food raised by irrigation of polluted water is often higher (Blayock, 2000). The major contributor to the accumulation of HMs in the food chain is supply sources. Ultimately, treatment of Pb sources prior to application to plants decreases the content of food crops. Different regions of global cereals and vegetables are irrigated with hazardous metal wastewater that poses serious health concerns. By simply avoiding inadequately treated waste water, pollution of HMs could be decreased by up to 85%. Another technique for reducing Pb content in food crops is land use optimization. Food crops should not be grown within 30 m of the roadside; urban roadside dust serves as a pathway for the accumulation of Pb in food crops (Uwah et al., 2011). Crops produced near the drainage area of acid mining have a high risk of contaminating Pb. Usually, contamination of Pb is from natural sources. Most researchers strongly support the use of pretreated wastewater for irrigation to decrease the content of HMs in food crops, including Pb. In addition, plant breeders and agronomists have to minimize soil crop transfer of Pb (Dong et al., 2009).

### Eco-Remediation

The prerequisite for remediation (eco-remediation) is a better understanding of soil-food crop transition mechanisms (Rai et al., 2019). By altering the physiochemical properties of the soil, these techniques minimize the bioavailability of HMs. The use of compost and biochar serves as an ecological solution to minimize the concentration of HMs in soil (as revealed in the measurement monitor model) by enhancing the ability of cation exchange, humification, and the cycle of soil nutrients. Biochar in combination with *Pseudomonas chenduensis* metal-resistant bacteria greatly decreases the bioavailability of HMs (Li et al., 2020). Detailed information on soil properties, such as biological and physiochemical characteristics and HM concentration, should be available for affective remediation. Here, we have a practical example in wheat plants grown hydroponically under Pb and nitric oxide (NO) supply. Researchers observed that NO significantly reduced the negative impact of Pb toxicity in the form of oxidative stress induced by ROS (malondialdehyde, conjugated dienes, hydroxyl ions and superoxide anion). Furthermore, exogenous NO interferes ROS detoxification mechanism and enhances the antioxidant enzyme activity in wheat roots (Kaur et al., 2015a). Based on the above results, eco-remediation technologies are therefore primarily applicable in the field of wet land agriculture. These approaches are cost-effective and environmentally friendly (Rai et al., 2019).

### Chemical and Physiochemical Strategies

Compared with biological strategies, chemical strategies are less preferable but are used to remediate the polluted soil. The complexion of metals in the soil makes it impossible for plants to have access to them. Synthetic zeolites with augmentation of alkaline clay are effectively used to remediate HMs (Chen et al., 2009). To recover soil from HMs, the use of red mud, magnetite, maghemite, silicon calcium fertilizer, hydrous manganese oxide, and hematite zeolite is productive. The soil plant system has the ability to cope with modifications to Pb, chalk, and manure to decrease both soil and H mobile components (Chandra et al., 2018). In the case of wheat, by using triple superphosphate, calcium magnesium phosphate, and single superphosphate in combination with ZnSO_4_, the phytoavailability of Pb (∼42.14%) in soils was decreased (Chemek et al., 2016). The activity of soil eco-enzymes and the protection of heavy metal concentration in cereal grains sown in chemical-treated industrial soil (acid washing/amendments) was the same as that grown on untreated soils; there were no risks to human health in both cases (Alamri et al., 2018).

Cereals like other plants have protective strategies against environmental contaminants like Pb by manipulating their genetically controlled chemical and physiochemical processes. These strategies operate in layers; firstly, by excluding or linking it to the cell wall, Pb entry into the cell is prevented; secondly, antioxidant production to fight ROS production is prevented (Rossato et al., 2012). The antioxidant protection mechanism was made up of various species such as carotenoids, glutathione, tocopherol, catalase, ascorbate, ascorbate peroxide, and superoxide dismutase found in vacuole (73%), chloroplast (17%), cytosole (5%), apoplast (4%), and 1% in mitochondria (Tangahu et al., 2011). Similarly, various proteins, including Pb, play an important role in counteracting the adverse effect of HMs (Mroczek-Zdyrska and Wójcik, 2012). The uptake of HMs such as Pb by plants is decreased and the uptake of nutritional elements such as Mg, Ca, Cu, and Fe is increased (He et al., 2004). In plants, Pb often initiates a protective mechanism against oxidative stress that relies entirely on the physiological status of plant organs (Rossato et al., 2012).

### Nanotechnology

A research hotspot of the present era is agro-nanotechnology; this ensures soil protection by reducing the bioavailability of HMs. The biosynthesis of nanoparticles is verified by advances in plant molecular biology techniques in protein and genetic engineering (Doshi et al., 2008). To detect the level of contamination in food crops, nanosensors are applied for analysis. In order to formulate less toxic biofertilizers and pesticides for the soil, different nanotechnologies may be used. Interaction between nanoparticles, plants, and soil prevails (Karami and Alipour, 2009). Owing to the accumulation of ambient particulate matter on the leaves of plants, there is a major shift in the mechanism of thermal equilibrium, photosynthesis, and transpiration. The suitable catalytic metal nanoparticles are limited to periodic Table Group VIII and IB (platinum, silver, gold, and palladium).

There is a need to know the level of penetration and transport of nanoparticles in plants to understand the role of nanotechnology in plant enhancement in stressful environments. Through its use in plant genomic analysis and gene function, nanotechnology can be used to enhance plant biological response to various types of environmental stresses (Monica and Cremonini, 2009). Nanoparticles are used to enhance germination, root growth, sources of micronutrients, the activities of Rubisco carboxilase and general plant growth in wheat and maize (Doshi et al., 2008).

**FIGURE 5:**
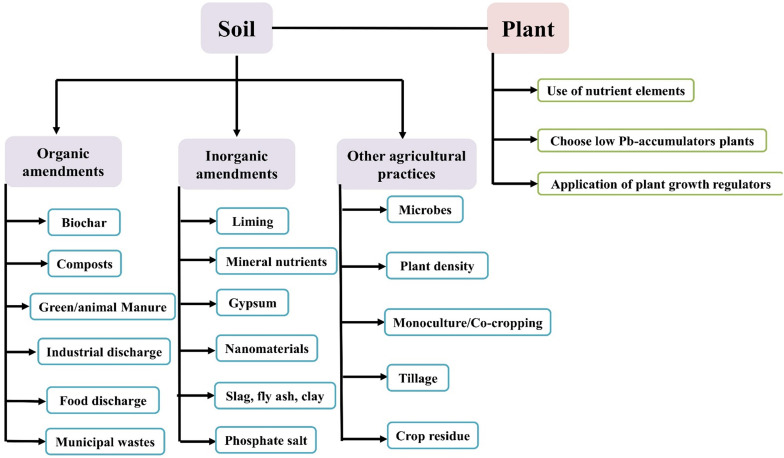
Phyto-management to reduce Pb uptake in cereals.

Electrochemical materials such as electrically conductive adhesives (ECA) exploration for surface mounting technology and flip chip application as Pb free alternatives were studied previously (Jiang et al., 2005; Kano et al., 2009; Karami and Alipour, 2009). Nanotechnological efforts are being carried out to increase plant resistance by improving the ability of plants to remove toxins from soil in order to use plants for soil clean-up (Figure 5).

## Effect on Genetic Characters

A direct measure of susceptibility or tolerance is the monitoring of biological responses of plants in the form of genetic characteristics such as genes, coding transcription factors (TF), and proteins responsive to environmental pollutants. The response of plants, including cereals, to external Pb stress relies on genotypic variation and concentration. Various cellular ingredients such as membrane stability/integrity, lipid peroxidation, redox potential assessment, metabolites (primary and secondary), and enzymatic and nonenzymatic antioxidants can be controlled to assess the oxidative (ROS) pressure caused by excessive Pb (Cassells and Curry, 2001). As biomarkers of Pb toxicity, these above-mentioned biochemicals and their regulated gene(s) expression are used to delineate effects long before symptoms emerge. Zhang et al. (2017) reported 262 differentially expressed TFs (DETFs) that react to Pb stress in maize plants. In addition, they showed that DETFs were divided into four classes. In addition, polymorphism at the level of expression of two bZIP TFs, such as ZmbZIP54 and ZmbZIP107, has been shown to contribute to the Pb tolerance mechanism in different lines of maize. Lead toxicity has significant effects on the pattern of gene expression. Many genes regulating various ingredients of plant metabolism are downregulated because of this form of stress, such as genes related to lipid metabolism, lignin biosynthesis, energy metabolism, xenobiotic biodegradation, metabolism of carbohydrates, metabolism of amino acids, metabolism of phenylalanine, and growth and death of cells. The upregulated genes that control characteristics are mostly linked to secondary metabolite biosynthesis, such as membrane transport, especially multidrug resistance protein, major facilitator superfamily, flavonoid biosynthesis, ascorbate and aldarate metabolism, ABC transporters, mitogen-activated protein kinases (MAPK) signaling pathway, glutathione metabolism, large numbers of GST, and relative water content. These are the key genetic variables to be used as select criteria for tolerance to Pb toxicity in cereals. Lead does not have a clear effect on the synthesis of chlorophyll *b*. Transcription variables play a very important role in plant responses to stresses from HMs, including Pb toxicity, and modulate various genes involved in these stresses. It also describes the variations in HM stresses in the network of pathways.

## Future Perspectives

A detailed study involving antioxidant mechanism, Pb-regulated differential gene expression, and mineral transporters is needed to understand complex plant responses to lead toxicity and other HMs. Different techniques such as flow cytometry (detection of changes in chromosome number and structure), fluorescent in situ hybridization (FISH), microdensitometry, restriction fragment length polymorphism (RFLP), and amplified fragment length polymorphism (AFLP) can be used to assess ROS-induced molecular level injuries. A more in-depth understanding of the stress caused by Pb toxicity in cereals by the use of biological markers would help to understand the mechanisms involved in Pb stress tolerance and improve cereal grain tolerance, which eventually help to increase the production of grain per unit area.

## Author Contributions

MA, AA, and RG designed and wrote the manuscript. MS, BA, ZU, UN, and WZ revised the manuscript. All authors contributed to the article and approved the submitted version.

## Conflict of Interest

The authors declare that the research was conducted in the absence of any commercial or financial relationships that could be construed as a potential conflict of interest.
